# Vitamin D supplementation to patients with frequent respiratory tract infections: a post hoc analysis of a randomized and placebo-controlled trial

**DOI:** 10.1186/s13104-015-1378-3

**Published:** 2015-08-30

**Authors:** Peter Bergman, Anna-Carin Norlin, Susanne Hansen, Linda Björkhem-Bergman

**Affiliations:** Division of Clinical Microbiology, Department of Laboratory Medicine, Karolinska Institutet and Karolinska University Hospital Huddinge, 141 86 Stockholm, Sweden; Division of Clincal Immunology, Karolinska Institutet and Karolinska University Hospital Huddinge, 141 86 Stockholm, Sweden; Infectious Disease Clinic, Karolinska University Hospital, 141 86 Stockholm, Sweden

**Keywords:** Vitamin D, Immunodeficiency, Respiratory tract infections, Infections, Supplementation, Randomized controlled trial

## Abstract

**Background:**

Vitamin D is considered to be important for a healthy immune system. The aim of this study was to test the hypothesis that vitamin D supplementation reduces number of respiratory tract infections (RTIs) and prolong the time to the first RTI in adult patients with frequent RTIs.

**Methods:**

We performed a post hoc analysis of a randomized, placebo-controlled and double-blinded study, where adult patients with a high burden of RTIs were randomized to placebo or vitamin D (4000 IE/day for 1 year, n = 124 in the per protocol cohort presented here).

**Results:**

Vitamin D supplementation increased the probability to stay free of RTI during the study year (RR 0.64, 95 % CI 0.43–0.94). Further, the total number of RTIs was also reduced in the vitamin D-group (86 RTIs) versus placebo (120 RTIs; p = 0.05). Finally, the time to the first RTI was significantly extended in the vitamin D-group (HR 1.68, 95 % CI 1.03–2.68, p = 0.0376).

**Conclusion:**

Vitamin D supplementation was found to significantly increase the probability of staying infection free during the study period. This finding further supports the notion that vitamin D-status should be monitored in adult patients with frequent RTIs and suggests that selected patients with vitamin D deficiency are supplemented. This could be a safe and cheap way to reduce RTIs and improve health in this vulnerable patient population.

The original trial was registered at http://www.clinicaltrials.gov (NCT01131858).

## Background

Vitamin D is considered to be important for a healthy immune system, by inducing expression of antimicrobial peptides in immune-cells and at epithelial surfaces [1]. In addition, vitamin D has broad anti-inflammatory effects on the adaptive immune system and can downregulate pro-inflammatory cytokines and increase immunoregulatory T-cells [2, 3]. Low levels of 25-hydroxyvitamin D (25-OHD) are associated with an increased risk of tuberculosis [4–6] and respiratory tract infections [7]. Previously, we have conducted a randomized, placebo controlled and double blind study where patients with primary immunodeficiency, with a high burden of infections, were randomized to placebo or vitamin D (4000 IE/day for 1 year). In this study (n = 124), vitamin D supplementation resulted in a significantly reduced infectious burden, compared to placebo. In addition, the total numbers of respiratory bacterial cultures were reduced by approximately 50 % and the antibiotic consumption was reduced by 60 % in the vitamin D-group [8].

The primary endpoint in the original study was a composite clinical score, based on a self-reported diary, as pre-specified in the original protocol. However, the protocol did not pre-define how an episode of acute respiratory infection (RTI) would be defined. Accordingly, this clinically relevant information was not reported in the original publication. Here we wanted to test the hypothesis that vitamin D treated patients had fewer RTIs and also had a longer time to their first RTI, compared to placebo treated patients. To this end we carried out a post hoc analysis of our previously performed study [8].

## Methods

### Study cohort

The study cohort consisted of the per protocol population (n = 124) from our previously performed clinical trial [8]. The original trial was a prospective, randomized and double-blinded placebo-controlled study of vitamin D_3_ supplementation to patients with primary immunodeficiency and increased susceptibility to respiratory tract infections, described in details elsewhere [8]. Patients were recruited at the Immunodeficiency Unit, Karolinska University Hospital, Huddinge, Sweden. Inclusion occurred between March and June, 2010. Inclusion criteria were 18–75 years of age and a documented increased susceptibility to respiratory tract infections, defined as >42 days with patient-reported symptoms of respiratory tract infection during a 12 months period prior to study inclusion. The patients’ diagnosis included selective IgA-deficiency (D80.2), IgG-subclass deficiency (D80.3) and common variable immune disorder (CVID, D83.0) as well as patients without a defined immunological diagnosis (D89.9). Exclusion criteria were prophylactic treatment with antibiotics, history of hypercalcaemia or stones in the urinary tract, sarcoidosis, ongoing supplementation with vitamin D3 exceeding 400 IU/day, HIV-infection and pregnancy. The raw data is accessed from the corresponding author upon request.

The intention to treat (ITT) population included n = 140 patients, who were randomized to 12 months’ treatment with oral vitamin D_3_ (Vigantol^®^, 4000 IU/day) or placebo oil (Miglyol). The per protocol population (n = 124), who completed the study, consisted of n = 62 vitamin D treated and n = 62 placebo treated patients.

The primary endpoint was pre-defined as “infectious score”, a composite score based on symptoms from the respiratory tract, ear, and nose as well as infectious malaise and antibiotic consumption. The patients filled out a questionnaire every day of the study period. Every symptom resulted in one point and the maximum score per day was 5 points. A day free of symptoms resulted in zero points. The questionnaire and full details of the results have been reported in the original publication [8].

To determine the number of RTI a thorough re-analysis of the original data-set was performed. One episode of RTI was defined as having “at least 2 points per day for at least 5 consecutive days”. If symptoms were zero or 1 point per day or did not last for 5 days or more, no RTI episode was recorded. The total number of RTIs was recorded for each patient and this information formed the basis for this post hoc analysis. In addition, the “time to first RTI” in days was also recorded and used for the Kaplan–Meier plot described in detail below. The time was defined as the number of days from study inclusion until the first episode with “at least 2 points per day for at least 5 consecutive days”.

### Ethical statement

The study was approved by the regional Ethical Review Board at Karolinska Institutet, Stockholm, Sweden and by the Swedish Medical Products Agency. The study was registered at http://www.clinicaltrials.gov (NCT01131858) prior to inclusion of the first patient (registered March 22, 2010 and approved May 26, 2010) and was conducted in accordance with the declaration of Helsinki. Written informed consent was obtained from all participants prior to inclusion.

### Statistical analysis

All statistical tests were performed using GraphPad Prism v. 6.00 and p < 0.05 were considered statistically significant.

#### Number of RTIs during the study

RTI was analysed in two different ways: first, this outcome was dichotomized (‘any RTI’ = 1 or more; and ‘zero RTI’) and analysed with Fishers exact test. Second, the total number of RTIs was analysed and due to a skewed distribution (many with zero RTIs), the non-parametric Mann–Whitney U test was applied.

#### Kaplan–Meier plot of time to first RTI

A survival analysis was performed to assess the time to first RTI in the two groups. The first RTI for all patients was connected to a certain day after inclusion. This information was plotted in a survival graph (Kaplan–Meier plot). Data was analyzed with the log rank Mantel Cox test and hazard ratio and the *p* value were calculated.

## Results

### Numbers of respiratory tract infections during the study

First, the total number of RTIs was analyzed in a dichotomized way (binary), i.e. patients were defined in two groups: having “no RTI” or “one or more RTI” (range 1–13 RTIs). Notably, a significantly lower fraction of patients in the vitamin D group had one or more RTI during the study (n = 26/62, 42 %), compared to n = 39/62 patients (63 %) in the placebo group (RR 0.64, 95 % CI 0.43–0.94, Fisher exact test).

Next, the total number of RTIs was studied on the group level (vitamin D/placebo). The 62 vitamin D patients documented 86 RTIs (median 0 RTI/patient, range 0–11), whereas the 62 placebo-patients reported 120 RTIs in total (median 1 RTI/patient, range 0–13) (p = 0.05, Mann–Whitney U test).

### Time to first acute respiratory tract infections

**Fig. 1:**
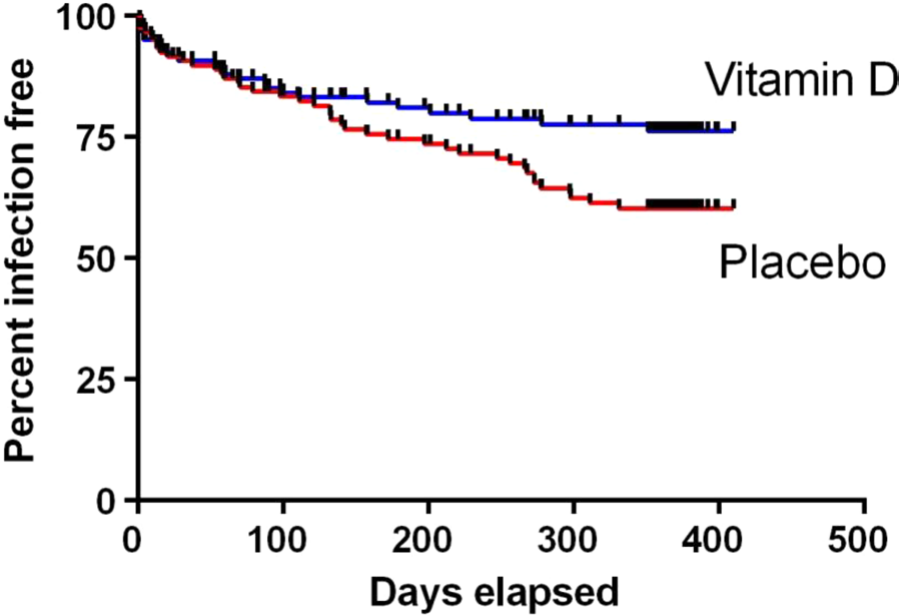
Time to first acute respiratory tract infection (RTI) in immunodeficient patients treated with vitamin D (4000 IE/day for 1 year) or placebo. There was a significantly higher probability to stay infection free in the vitamin D-group (HR 1.68, 95 % CI 1.03–2.68, p = 0.038, Log rank Mantel Cox test)

The time to first RTI was also identified based on analyses of the original dataset. This information was used to perform Cox-analysis between the two groups. Interestingly, the first 120 days, there was no difference between the two groups, but after this time the vitamin D group clearly had a better outcome with regard to RTI than the placebo group (HR 1.68, 95 % CI 1.03–2.68, p = 0.038, Log rank Mantel Cox test, Fig. 1).

## Discussion

Here we tested the hypothesis that vitamin D supplementation could reduce the number of—and extend the time to—the first RTI, in a group of patients with increased susceptibility to RTIs (with and without antibody deficiency). To test this hypothesis we re-analyzed the full dataset from the original study and defined the criteria for an ‘RTI-event’ to be “at least 2 points per day for at least 5 consecutive days”. By applying this model, we could obtain information on the number of RTIs and the time to first RTI. This information formed the basis for the current report. Notably, vitamin D supplementation appeared to decrease the number of RTIs and also prolonged the time to first RTI.

One interesting finding was that the beneficial effect of vitamin D supplementation appeared after approximately 3 months (120 days). Notably, this coincided with significantly increased serum-levels of 25-OH vitamin D in the supplemented individuals, as described in the original publication [8], thus strengthening a relationship between 25-OH vitamin D levels and RTIs.

The main advantage of redefining the infectious score to RTI, is the generalizability of the results, i.e. that the results can be compared with other studies. In addition, the results are easier to communicate to physicians and patients. Although the “infectious score” represents most main aspects of having an RTI (symptoms from the respiratory tract, ears or sinuses as well as infectious malaise and antibiotic consumption), it is a composite score, which may be difficult to translate into a clinically meaningful result. In contrast, “the numbers of RTI” and “time to first RTI”, are two outcome parameters, which clearly have meaning for doctors and health care policy-makers.

Importantly, these results stem from a randomized controlled trial (RCT), and carry all the strengths inherent to such a design, including randomization, blinding and lack of residual bias.

However, it is important to state that the current study is a post hoc analysis of an already published trial [8]. As such, the definition of an RTI-event was not predefined in the original protocol. Nevertheless, all information to calculate the number of RTI and time to RTI was collected during the primary trial. Thus, no additional data collection has occurred. It should also be noted that the current analysis is performed per protocol and not as intention to treat. However, we believe that the per protocol analysis represent the data in a valid manner, since there was an equal amount of patients excluded from each arm. The reasons for exclusion were mainly due to lack of compliance to the protocol, i.e. not taking the drug or not filling out the diary. Importantly, there was no tendency that any reason for exclusion was associated with a specific allocation (vitamin D/placebo), thus minimizing the risk of bias in a per protocol analysis.

The role of vitamin D in respiratory tract infections is still not clear, despite several large RCTs in the area (reviewed in [9]). This can probably be explained by the large heterogeneity in these RCTs. For example, they differ with regard to study population (healthy/disease), geographical location (north/south), baseline vitamin D levels (deficient or not), dose of vitamin D (high/low), dosage regimen (daily/bolus) or study length (months/years). All these aspects most likely have an impact on the outcome of the respective study. Interestingly, our study did find a beneficial effect of vitamin D on respiratory tract infection, both in the previous trial (endpoint infectious score) [8] but also here (number of RTI, time to first RTI). There are several reasons for why our study had a positive outcome. For example, we studied patients with frequent RTIs (at least 42 days with infectious symptoms the year prior to inclusion). Further, the baseline vitamin D levels were quite low (median 50 nmol/L) and few had levels above 75 nmol/L (11 %) or 100 nmol/L (4 %). This is in contrast to the VIDAS-trial from New Zealand, with a null result, where median levels at inclusion were 75 nmol/L. In addition, we used a daily dosing schedule, which have been proposed to be superior to a bolus schedule (monthly dosing), although this remains to be unequivocally proven [9].

Our study also had several important limitations. First, the definition of a RTI-episode that we chose did not take the severity into account. We used “at least 2 points for at least 5 consecutive days” as a definition, where 1 point could involve symptoms from the respiratory tract, sinuses or ears or malaise or antibiotics. With this definition one severe RTI with all symptoms + antibiotics for a prolonged period would still only count as one RTI. This is of course a limitation, but we think that the severity of RTIs was well covered in the original article where the “infectious score” was used as the primary endpoint. Here, the aim was to obtain a clinically useful outcome and thus we chose “number of RTI” and “time to first RTI” as endpoints. Another limitation is that the study population was a selected group of patients who are registered at our tertiary university hospital clinic, with various immunological disorders. Thus, the results described here cannot be extrapolated to other patient groups or to the general public.

The data presented here provide additional evidence for a beneficial role of vitamin D in preventing RTIs. Although many pieces of key information are still lacking, including the dose, target vitamin D serum levels and populations in need of supplementation, we believe that vitamin D should be a parameter to be taken into account when meeting patients with frequent respiratory tract infections. It is also important to say that we did not record any related adverse events (AE); in fact for several types of AEs, there were fewer events recorded for the vitamin D group, compared to the placebo treated patients [8]. Thus, vitamin D represents a safe, cheap and potentially important factor to correct among vitamin D deficient patients with frequent RTIs.

Given the fact that published RCTs in the area are heterogeneous and not conclusive, additional RCTs are warranted. These new RCTs should aim to define the correct dose, the adequate target vitamin D serum level to prevent RTIs and be performed in different disease groups, including chronic obstructive pulmonary disease (COPD), chronic sinusitis and chronic lung-disease.

## Conclusion

In this post hoc analysis of a randomized clinical trial vitamin D supplementation was found to significantly increase the probability of staying infection free during the study period. This finding further support the notion that vitamin D-status should be monitored in patients with frequent RTIs and that selected patients with vitamin D-deficiency are supplemented. This could be a safe and cheap way to reduce RTIs and improve health in this vulnerable patient population.

## Abbreviations

RTI: respiratory tract infection
CI: confidence interval
25OHD: 25-hydroxy vitamin D_3_
HR: hazard ratio
RCT: randomised controlled trial
AE: adverse event
COPD: chronic obstructive pulmonary disorder
RR: risk ratio
